# NAT10-mediated ac^4^C acetylation of TFRC promotes sepsis-induced pulmonary injury through regulating ferroptosis

**DOI:** 10.1186/s10020-024-00912-w

**Published:** 2024-09-09

**Authors:** Pengcheng Xing, Minjie Zhou, Jian Sun, Donglian Wang, Weipeng Huang, Peng An

**Affiliations:** Department of Emergency and Intensive Care Unit, Shanghai Sixth People’s Hospital Affiliated to Shanghai Jiao Tong University School of Medicine, No. 222, West Three Road Aroud Lake, Nanhui New Town, Pudong New Area, Shanghai, 201306 China

**Keywords:** Sepsis-induced pulmonary injury, NAT10, Ac^4^C, TFRC, Ferroptosis

## Abstract

**Background:**

Sepsis-induced pulmonary injury (SPI) is a common complication of sepsis with a high rate of mortality. N4-acetylcytidine (ac^4^C) is mediated by the ac^4^C “writer”, N-acetyltransferase (NAT)10, to regulate the stabilization of mRNA. This study aimed to investigate the role of NAT10 in SPI and the underlying mechanism.

**Methods:**

Twenty-three acute respiratory distress syndrome (ARDS) patients and 27 non-ARDS volunteers were recruited. A sepsis rat model was established. Reverse transcription-quantitative polymerase chain reaction was used to detect the expression of NAT10 and transferrin receptor (TFRC). Cell viability was detected by cell counting kit-8. The levels of Fe^2+^, glutathione, and malondialdehyde were assessed by commercial kits. Lipid reactive oxygen species production was measured by flow cytometric analysis. Western blot was used to detect ferroptosis-related protein levels. Haematoxylin & eosin staining was performed to observe the pulmonary pathological symptoms.

**Results:**

The results showed that NAT10 was increased in ARDS patients and lipopolysaccharide-treated human lung microvascular endothelial cell line-5a (HULEC-5a) cells. NAT10 inhibition increased cell viability and decreased ferroptosis in HULEC-5a cells. TFRC was a downstream regulatory target of NAT10-mediated ac^4^C acetylation. Overexpression of TFRC decreased cell viability and promoted ferroptosis. In in vivo study, NAT10 inhibition alleviated SPI.

**Conclusion:**

NAT10-mediated ac^4^C acetylation of TFRC aggravated SPI through promoting ferroptosis.

**Graphical Abstract:**

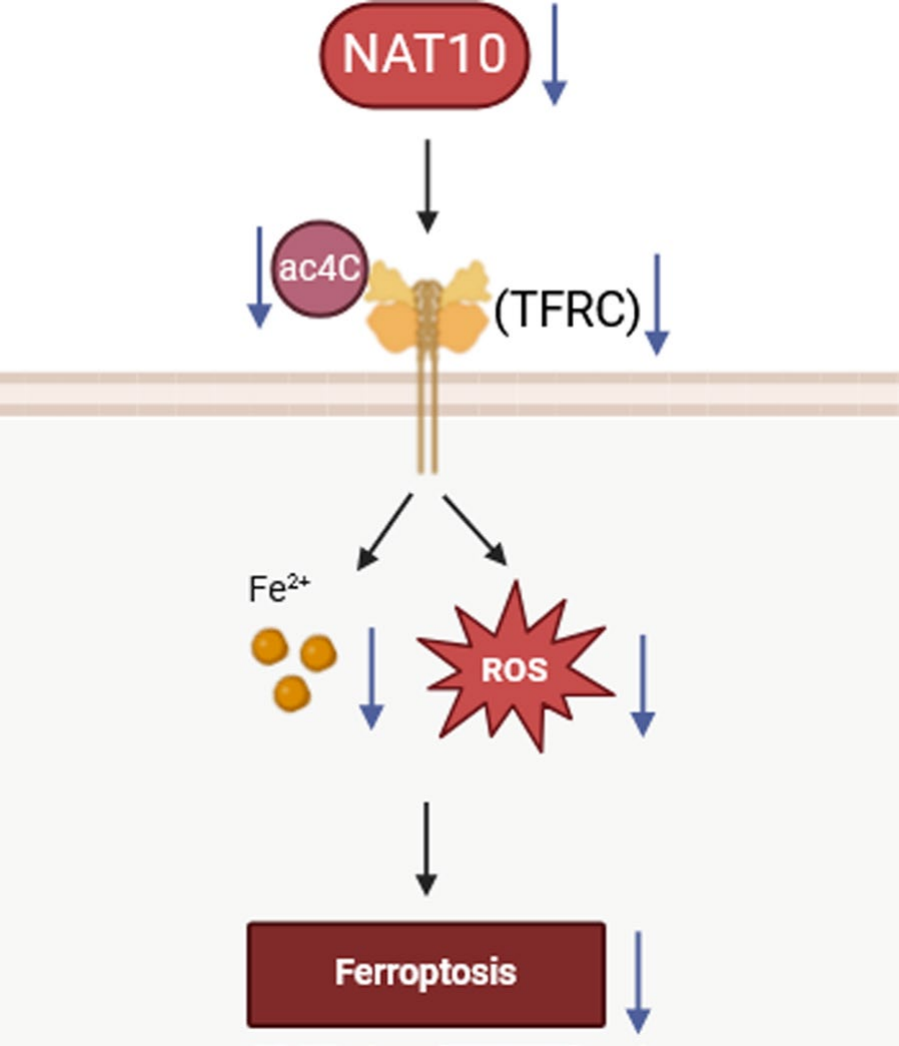

## Introduction

Sepsis is defined as a life-threatening organ dysfunction caused by a dysfunctional host response to infection (Evans et al. 2021). Lung is the most vulnerable target organ in sepsis (Kocak et al. 2021; Cribbs et al. 2010). Acute lung injury (ALI), one of the most common complications of sepsis, is difficult to treat and has a high fatality rate (Zhou and Liao 2021; Li et al. 2022a). ALI is characterized by decreased lung volume and compliance and dysregulated ventilation/blood flow ratio. The clinical manifestations of ALI are diffuse alveolar injury, progressive hypoxemia, and respiratory distress, and the pulmonary imaging findings were heterogeneous exudative lesions. Acute respiratory distress syndrome (ARDS) is the severe form of ALI with an oxygenation index of less than 200 (Fan and Fan 2018). At present, the pathogenesis of sepsis-associated ALI/ARDS is still unclear, and it may be the result of multiple factors, including inflammation, pulmonary surfactant, oxidant stress, and pyroptosis. In addition, epigenetic modifications including N6-methyladenosine (m^6^A) and N4-acetylcytidine (ac^4^C) may also be an important mechanism affecting the occurrence and development of sepsis-induced pulmonary injury (SPI) (Zhang et al. 2023, 2022a).

The mRNA and non-coding RNA in cells contain a large number of apparent chemical modifications. Ac^4^C is a modification of cytidine at the nitrogen-4 site and plays an important role in mRNA translation (Lou et al. 2023). Previous research suggests that ac^4^C is essential for cellular mRNA stability, rRNA biosynthesis, cell proliferation, epithelial-mesenchymal transition, tumor development, and drug resistance (Xie et al. 2023; Jin et al. 2020; Wei et al. 2023). NAT10, a nuclear protein with lysine acetyltransferase action, is regarded as an ac^4^C “writer”, which is involved in a variety of cellular processes (Xie et al. 2023). Various studies imply that NAT10 regulates the expression of target genes and the biological functions of various cancers by mediating ac^4^C acetylation (Wei et al. 2023; Pan et al. 2023). A previous study reveals that downregulation of NAT10 exacerbates pyroptosis, thus promoting the progression of sepsis (Zhang et al. 2022a). In addition, NAT10 acts as a regulator of the ferroptosis pathway in many diseases (Dalhat et al. 2023; Shen et al. 2024). However, the regulation of NAT10 in SPI has been rarely reported.

Ferroptosis, first reported in 2012 by Dixon (Dixon et al. 2012), is a novel regulatory mode of cell death mediated by lipid peroxides and lipid reactive oxygen species (ROS) accumulation, and its pathophysiological regulatory mechanism is complex. According to the published literature, the main known pathways of ferroptosis include glutathione (GSH)/glutathione peroxidase 4 (GPX4) pathway, mevalonic acid pathway, iron accumulation, and lipid peroxidation (Zhang et al. 2021; Sha et al. 2021; Rochette et al. 2022). Normally, the lungs maintain iron metabolic homeostasis and protect the lungs from oxidative stress through macrophages, transferrin, and lung epithelial cells. However, endogenous or exogenous factors lead to the imbalance of iron homeostasis in the lung, resulting in excess iron, which then leads to the formation of lipid ROS through Fenton reaction, causing ferroptosis and aggravating pulmonary injury. The role of ferroptosis in SPI has been reported before (Zhang et al. 2022b). Whereas, the effects of NAT10 on ferroptosis in SPI has not been investigated.

Given this background, we hypothesized that NAT10-mediated ac^4^C acetylation promoted SPI through regulating ferroptosis. This study may provide novel targets for SPI therapy.

## Methods and materials

### Clinical study

Lung tissue specimens from 23 cases of ARDS patients and 27 healthy volunteers (non-ARDS) in Shanghai Sixth People’s Hospital Affiliated to Shanghai Jiao Tong University School of Medicine were collected and stored in liquid nitrogen for use. All tissue samples were verified by pathological examination. Patients signed informed consent. This study was approved by Shanghai Sixth People’s Hospital Affiliated to Shanghai Jiao Tong University School of Medicine. Basic information of all patients and volunteers was collected and shown in Table 1.

**Table 1:** General characteristics of ARDS patients and Non-ARDS volunteers

Variable	Non-ARDS (n = 27)	ARDS (n = 23)	*p* value
Age (years)	60.85 ± 9.30	61.83 ± 7.00	0.6818
Gender			0.7412
Male	14	13	
Female	13	10	
BMI (kg/m^2^)	23.53 ± 3.09	24.12 ± 3.69	0.5373
Smoking, No. (%)			
Current	8 (29.62%)	15 (62.50%)	
Former	9 (33.33%)	2 (8.70%)	
Never	10 (37.05%)	6 (28.80%)	
CRP (mg/L)	5.25 ± 1.49	174.80 ± 27.70	0.009
PCT (ng/ml)	0.037 ± 0.008	3.703 ± 0.315	< 0.001

### Cell culture

Human lung microvascular endothelial cell line-5a (HULEC-5a) was purchased from American Type Culture Collection (ATCC) and cultured with Dulbecco’s modified eagle medium (DMEM; Gibco, New York, USA) containing 10% fetal bovine serum (FBS; Gibco) and 1% penicillin and streptomycin (FBS; Gibco). The cells were cultured in an incubator with 37 ℃ and 5% CO_2_.

### Cell transfection and treatment

Negative control pcDNA 3.1 vector, pcDNA 3.1-TFRC overexpression vector, short hairpin (sh) negative control (sh-NC) plasmid, and sh-NAT10 plasmid were synthesized by Genomeditech Biotechnology Co., LTD (Shanghai, China). HULEC-5a cells (5 × 10^5^ cells/well) were inoculated in 6-well plates (Corning, NY, USA). After the cell confluence reached 80%, transfection was performed for 48 h using Lipofectamine 3000 (Thermo Fisher Scientific; Waltham, MA, USA).

Besides, HULEC-5a cells were stimulated with lipopolysaccharide (LPS; Sigma-Aldrich, St. Louis, MO,USA; 1 μg/mL) for 24 h (Wang et al. 2021), tauroursodeoxycholic acid (TUDCA; 500 mM; Yeason Biotechnology Co., LTD, Shanghai, China) for 12 h, necrosulfonamide (NSA; 20 μmol/L; Abcam, Cambridge, MA, USA) for 2 h, and ferrostatin-1 (Fer-1; 5 μM; Sigma) for 36 h.

### Western blot

Cells were lysed using the RIPA lysis buffer (Thermo Fisher) on ice to extract proteins. After detecting protein concentration using the BCA method (Sigma), 50 μg of the proteins were loaded on 10% sodium dodecyl sulfate–polyacrylamide gel electrophoresis (SDS-PAGE) for separation. Then, the proteins were transferred to the PVDF membrane (Sigma). The membrane was blocked in western blocking buffer (Thermo Fisher) for 1.5 h at room temperature, and incubated with the primary antibodies overnight at 4 °C for immunoblotting. The primary antibodies used were rabbit antibodies specific for GPX4 (1/1000; ab125066; Abcam), solute carrier family 7 member 11 (SLC7A11; 1 µg/mL; 711,589; Thermo Fisher), achaete-scute complexlike 4 (ACSL4; 1/1000; PA5-27,137; Thermo Fisher), and glyceraldehyde-3-phosphate dehydrogenase (GAPDH; 1/1000; A-11008; Thermo Fisher). Then, the membrane was washed thrice by Tris-buffered saline Tween (TBST; Yeason), and incubated with the secondary antibody (1/10000; 31,460; Thermo Fisher) for 1 h at room temperature. Finally, the membranes were washed with TBST and a SuperPico ECL Chemiluminescence kit (Vazyme Biotechnology Co., LTD, Nanjing, China) was used to expose the protein bands.

### Reverse transcription-quantitative polymerase chain reaction (RT-qPCR)

Total RNA from cells and tissues was extracted using Trizol regent (Yeason). The quality of isolated RNA was measured with a Nanodrop 2000 spectrophotometer (Thermo Fisher) and the RNA concentration was adjusted to 500 ng/μL. To obtain cDNA, the reverse transcription were carried out using the PrimeScript RT reagent Kit (Takara, Tokyo, Japan), and the qPCR amplification experiment was performed using the TB Green® Premix Ex Taq II FAST qPCR (Takara) with the provided reaction conditions. All primers used in this study were synthesized by Genescript Biotechnology Co., LTD, (Nanjing, China) and listed as follows: N-acetyltransferase (NAT)10, forward, 5′-ATAGCAGCCACAAACATTCGC-3′ and reverse, 5′-ACACACATGCCGAAGGTATTG-3′; transferrin receptor (TFRC), forward, 5′-ACCATTGTCATATACCCGGTTCA-3′ and reverse, 5′-CAATAGCCCAAGTAGCCAATCAT-3′; glyceraldehyde-3-phosphate dehydrogenase (GAPDH), 5′-TGTGGGCATCAATGGATTTGG-3′ and reverse, 5′-ACACCATGTATTCCGGGTCAAT-3′. The gene expression was calculated by the 2^−ΔΔCT^ method and GAPDH was used as the internal control.

### Cell counting kit-8 (CCK-8) assay

The cells were first seeded into a 96-well plate at the density of 1 × 10^3^ cells/well. Then, the cells were maintained in the incubator for 24 h, and 10 μL of CCK-8 solution (Vazyme) was added to each well to incubate with cells for 2 h. A microplate reader (Thermo Fisher) was used to assess the absorbance at 450 nm. Three replicate wells were set up.

### Determination of cellular ferrous iron (Fe^2+^), malondialdehyde (MDA), and GSH

Fe^2+^ level, MDA content, and GSH concentration in cells or tissues were detected by commercial Ferrous Ion Content Assay (Solarbio, Beijing, China), MDA Assay (Jiancheng Biotechnology Co. Ltd., Nanjing, China), and GSH Assay kits (Jiancheng) according to the provided instructions.

### Flow cytometric analysis of lipid ROS production

ROS production detection was performed as previously described (Jiang et al. 2022). Cells were incubated with the Molecular Probes BODIPY 581/591C11 (Invitrogen, Carlsbad, CA, USA) working solution (5 μmol/L) at 37 °C for 30 min without light. Next, the cells were washed with phosphate buffer solution three times and measured using flow cytometric analysis.

### ac^4^C-RNA immunoprecipitation (RIP)

The ac^4^C-RIP assay was performed according to the published literature (Chen et al. 2023). Briefly, RNA fragments were incubated with an anti-ac^4^C antibody (Abcam, ab252215) or IgG (Abcam, ab172730), and ac^4^C-modified RNA was then eluted for ac^4^C enrichment analysis by qPCR.

### RIP assay

RIP assay was used to explore the interaction between TFRC and NAT10 in HULEC-5a cells using the Imprint RIP Kit (Merck Millipore, Billerica, MA, USA). In brief, cells were lysed in RIP buffer for 30 min at 4 °C. Then, the cell supernatant was incubated with magnetic beads (Merck Millipore) bound with IgG and NAT10 antibodies (Abcam) for 4 h at 4 °C. After incubation, the beads were washed and eluted. Then, proteinase K was added to remove protein at 55 °C for 30 min. The RNA was isolated and qPCR was performed to detect TFRC expression.

### Dual-luciferase reporter assay

The cDNA containing full-length 3'-untranslated regions (UTR) of FTRC was cloned into the pGL3 luciferase reporter vector (Promega, Madison, WI,USA) to obtain pGL3-FTRC-wild type (WT). Besides, pGL3-FTRC-mutant type (MUT) was obtained by the introduction of mutations into pGL3-FTRC-WT using the QuikChange II Site-Directed Mutagenesis kit (Agilent Technologies, Santa Clara, CA, USA). Next, sh-NC/sh-NAT10 and pGL3-FTRC-WT/pGL3-FTRC-MUT were co-transfected into HULEC-5a cells using Lipofectamine 3000 for 48 h. Afterwards, luciferase activity was measured using Dual-Luciferase® Reporter Assay System Kit (Promega) and normalized to the activity of *Renilla* luciferase. The ratio of firefly/*Renilla* luciferase activity was used as the relative luciferase activity.

### RNA stability assessment

RNA stability assessment was performed to verify the stability of TFRC after NAT10 silenced in HULEC-5a cells. HULEC-5a cells were treated with actinomycin (Act) D (0.5 µg/mL, Yeason), then existing TFRC expression at different time points (1, 4, 8, and 12 h) was analyzed by qPCR.

### Animal study

A total of 24 male Wistar (6–8 weeks old) rats were purchased from Charles River (Beijing, China) and housed in cages with 24 ℃, a 12 h alternating light/dark cycle and free access to water and food. After one-week adaptive feeding, the rats were randomly divided into four groups (n = 6 per group): sham, cecum ligation and puncture (CLP), CLP + sh-NC, and CLP + sh-NAT10 groups.

The sepsis rat model was established according to a previous study (Li et al. 2022b). Briefly, rats were anesthetized with 2% sodium pentobarbital (0.3 mL/100 g; Sigma) by abdominal cavity injection after fasting for 12 h. Next, median abdomen hair of the rats was scraped and disinfected using iodine and medical alcohol. Incision was made (about 2 cm) along the linea alba to locate the cecum and ligation with 5–0 suture at about 1/3 of the cecum. The cecum was punctured with a needle, ligated twice. A small amount of intestinal contents were squeezed out of the puncture hole to ensure that the cecum is unobstructed. Next, the treated cecum was reintroduced into the abdominal cavity and the inner layer was sutured with 5–0 thread, and the outer layer, with 3–0 thread. Finally, the rats were placed on a heating pad and the rectal temperature was maintained at 37 ± 0.5 °C. When the rats woke up, they were fed and drank freely. The sham group rats received the same operation except cecal ligation and puncture. For NAT10 knockdown, lentivirus containing sh-NAT10 and sh-NC (0.2 mL, 1 × 10^9^ pfu/mL) were injected into the caudal vein 4 days before modeling, respectively. Finally, all rats were sacrificed by administration of an overdose of anesthesia of sodium pentobarbital. The lung tissues from each rat were collected and stored at − 80 °C for subsequent experiments.

### Hematoxylin & eosin (H&E) staining

The lung tissues were fixed with 4% paraformaldehyde (Sigma). Next, the samples were embedded with paraffin. After that, the tissue sections were cut into 5 μm and stained with hematoxylin and eosin (Yeason) for 5 min. Finally, the sections were observed by a light microscope.

### Statistical analysis

The SPSS 21.0 software was used to analyze data. Data are expressed as mean ± standard deviation (SD). Student’s t-test was used for comparison between the two groups. One-way analysis of variance (ANOVA) with Tukey’s post hoc analysis was used for comparison among groups. R language and HemI 1.0 software (http://hemi.biocuckoo.org/index.php) were used to generate a heatmap showing the expression of ferroptosis-related genes in sh-NC and sh-NAT10 groups. Potential diagnostic value of NAT10 in SPI was presented by receiver operating characteristics (ROC) curve analysis. Statistical analyses were performed using GraphPad Prism software (v8.0.1, GraphPad Software Inc., San Diego, CA, USA). *p* < 0.05 indicates that the difference is statistically significant.

## Results

### NAT10 was increased in ARDS patients and LPS-treated HULEC-5a cells

The basic information of ARDS patients and non-ARDS volunteers was listed in Table 1. The results indicated that the age, gender, and body mass index (BMI) between two groups showed no significant differences. Besides, compared with the non-ARDS group, ARDS patient showed higher C-reaction protein (CRP) level and procalcitonin (PCT) concentration.

**Fig. 1:**
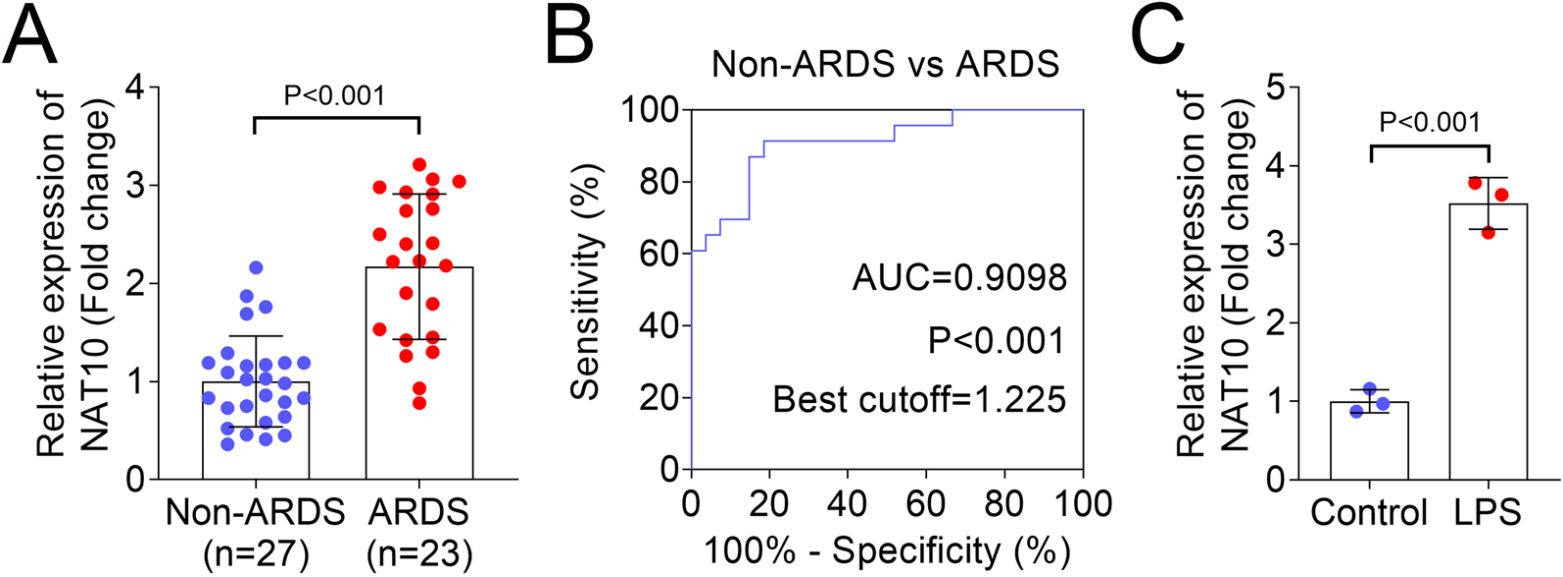
NAT10 was increased in ARDS patients and LPS-treated HULEC-5a cells. **A** RT-qPCR was performed to evaluate the expression of NAT10 in non-ARDS and ARDS groups; **B** The ROC curves of NAT10 in non-ARDS and ARDS groups; **C** The level of NAT10 in control and LPS groups was detected by RT-qPCR. NAT10: N-acetyltransferase 10; LPS: lipopolysaccharide; RT-qPCR: reverse transcription-quantitative polymerase chain reaction; ARDS: acute respiratory distress syndrome; ROC: receiver operating characteristic

The regulatory role of NAT10 in sepsis has been reported before (Zhang et al. 2022a). However, the role of NAT10 in SPI is unclear. In our study, we recruited 23 ARDS patients and 27 non-ARDS volunteers. We found that compared with the volunteers, the lung tissues of ARDS patients showed higher NAT10 expression (Fig. 1A). Next, ROC curve analysis was conducted to evaluate the sensitivity and specificity of NAT10 in ARDS. The area under the curve (AUC) is characterized by sensitivity and specificity and is often used to indicate the intrinsic validity of a diagnostic test (Kumar and Indrayan 2011). Based on the ROC curve, the optimal cutoff in the expression of NAT10 for detecting ARDS was 1.225 (Youden index: 0.728). NAT10 (AUC = 0.9098) showed a good prediction of ARDS (Fig. 1B). In further in vitro study, the results indicated that LPS treatment increased the mRNA level of NAT10 compared with the control group (Fig. 1C).

### Silencing of NAT10 increased cell viability and inhibited ferroptosis in HULEC-5a cells

**Fig. 2:**
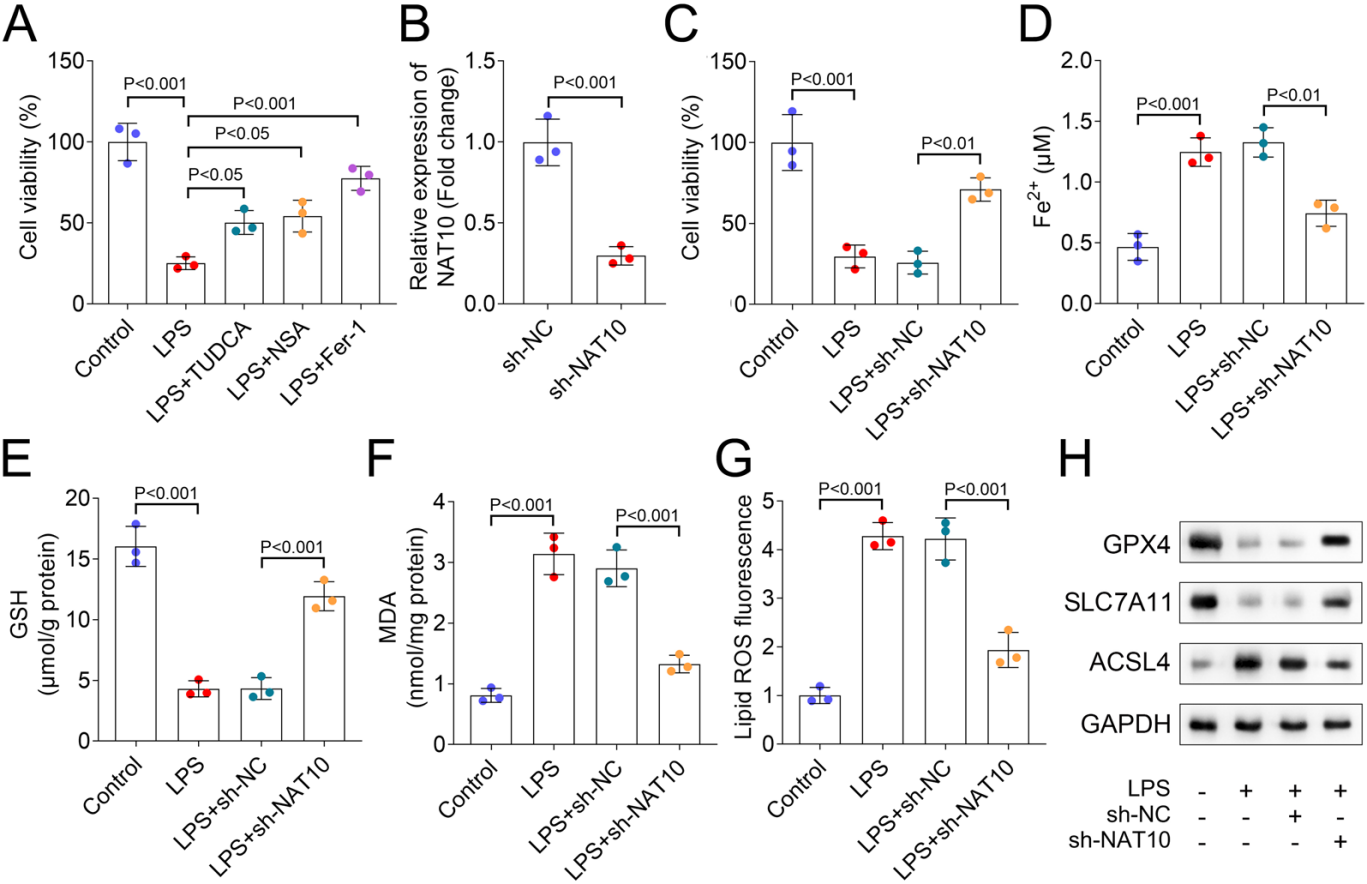
Silencing of NAT10 increased cell viability and inhibited ferroptosis in HULEC-5a cells. **A** CCK-8 assay was used to assess the cell viability of HULEC-5a cells in each group; **B** The expression of NAT10 after NAT10 knockdown was detected by RT-qPCR; **C** The cell viability in each group was analyzed by CCK-8 assay; **D** Fe^2+^, **E** GSH, and **F** MDA concentrations in each group were analyzed by commercial kits; **G** Flow cytometric analysis of fluorescence intensity; **H** Western blot was performed to assess the protein levels of GPX4, SLC7A11, and ASCL4 in each group. CCK-8: cell counting kit-8; RT-qPCR: reverse transcription-polymerase chain reaction; HULEC-5a: human lung microvascular endothelial cell line-5a; GSH: glutathione; MDA: malondialdehyde; GPX4: glutathione peroxidase 4; SLC7A11: solute carrier family 7 member 11; ASCL4: achaete-scute complexlike 4

TUDCA is an inhibitor of endoplasmic reticulum stress, significantly reduces the expression of apoptotic molecules, and is widely involved in the progression of various diseases such as liver injury and neurodegenerative disorders (Zangerolamo et al. 2021; Sun et al. 2020). NSA, a drug that inhibits cell death and pyrosis, works by acting on mixed lineage kinase domain-like protein (Yang et al. 2022). Fer-1, a ferroptosis inhibitor, shows protective roles in sepsis (Xiao et al. 2021). In our study, we found that LPS group showed decreased cell viability in comparison with the control group. Besides, compared with the LPS group, cell viability in TUDCA, NSA, and Fer-1 were increased (Fig. 2A). Compared with NSA and TUDCA treatment, the treatment of Fer-1 improved the cell viability after LPS treatment more significantly, thus we wanted to further investigate the role of ferroptosis in LPS-treated HULEC-5a cells. We transfected sh-NC and sh-NAT10 plasmids into HULEC-5a cells. Compared with the sh-NC group, silencing of NAT10 showed inhibited NAT10 expression (Fig. 2B). Besides, compared with the sh-NC group, NAT10 inhibition increased cell viability (Fig. 2C). Ferroptosis-related indicators results showed that compared with the control group, LPS treatment increased Fe^2+^ level, decreased GSH content, and upregulated MDA and lipid ROS fluorescence. In comparison with the sh-NC group, NAT10 knockdown inhibited Fe^2+^ level, MDA content, and lipid ROS fluorescence, and increased GSH concentration in HULEC-5a cells (Fig. 2D–G). Western blot results revealed that compared with the control group, LPS group showed decreased GPX4 and SLC7A11 protein levels and increased ACSL4 protein level. In addition, compared with the sh-NC group, NAT10 inhibition increased GPX4 and SLC7A11 protein levels and decreased that of ACSL4 in HULEC-5a cells (Fig. 2H).

### TFRC was a downstream regulatory target of NAT10-mediated ac^4^C acetylation in HULEC-5a cells

**Fig. 3:**
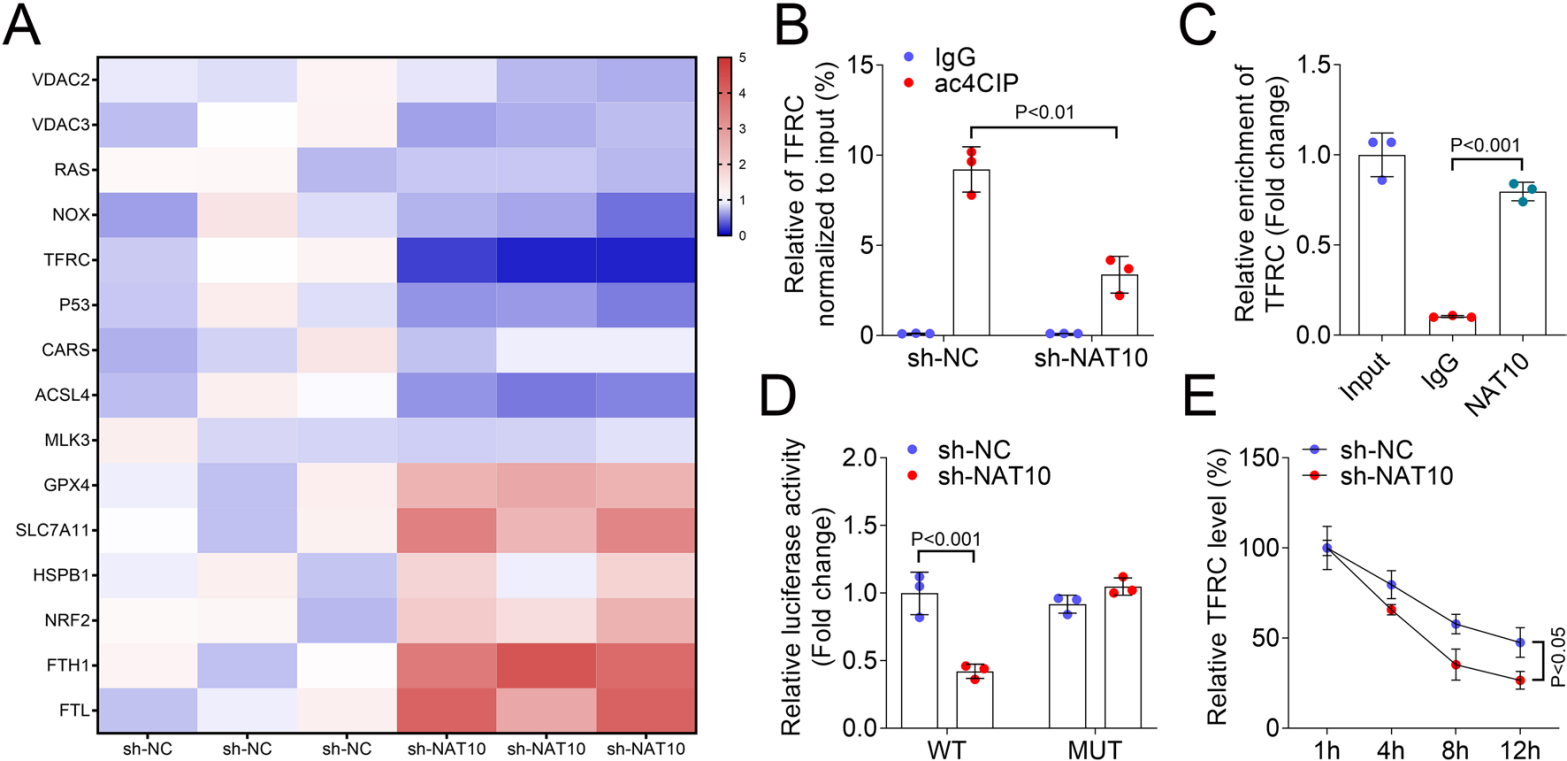
TFRC was a downstream regulatory target of NAT10-mediated ac^4^C acetylation in HULEC-5a cells. **A** Heatmap showed the expression of ferroptosis-related genes in sh-NC and sh-NAT10 groups; **B** The ac^4^C-RIP assay revealed the ac^4^C abundance on TFRC mRNA in sh-NC and sh-NAT10 groups; **C** RIP assay revealed the interaction between NAT10 and TFRC in HULEC-5a cells; **D** Dual-luciferase reporter assay evaluated the binding of NAT10 and TFRC; **E** RNA stability assay was used to detect the existing TFRC expression when actinomycin D treated at different time points (1, 4, 8, and 12 h). NAT10: N-acetyltransferase 10; ac^4^C: N4-acetylcytidine; TFRC: transferrin receptor; RIP: RNA immunoprecipitation; qPCR: quantitative polymerase chain reaction

The heatmap was used to show the expression of ferroptosis-related mRNA in sh-NC and sh-NAT10 groups. The results showed that TFRC decreased most significantly when NAT10 was silenced (Fig. 3A). Additionally, ac^4^C-RIP assay indicated that inhibition of NAT10 showed a lower level of ac^4^C of TFRC mRNA in HULEC-5a cells (Fig. 3B). RIP assay indicated that NAT10 bound with the mRNA of TFRC (Fig. 3C). Dual-luciferase reporter assays showed that NAT10 was specifically bound to TFRC (Fig. 3D). RNA stability assessment revealed that silencing of NAT10 resulted an accelerated degradation of TFRC mRNA in HULEC-5a cells (Fig. 3E).

### Overexpression of TFRC decreased cell viability and promoted ferroptosis in HULEC-5a cells

**Fig. 4:**
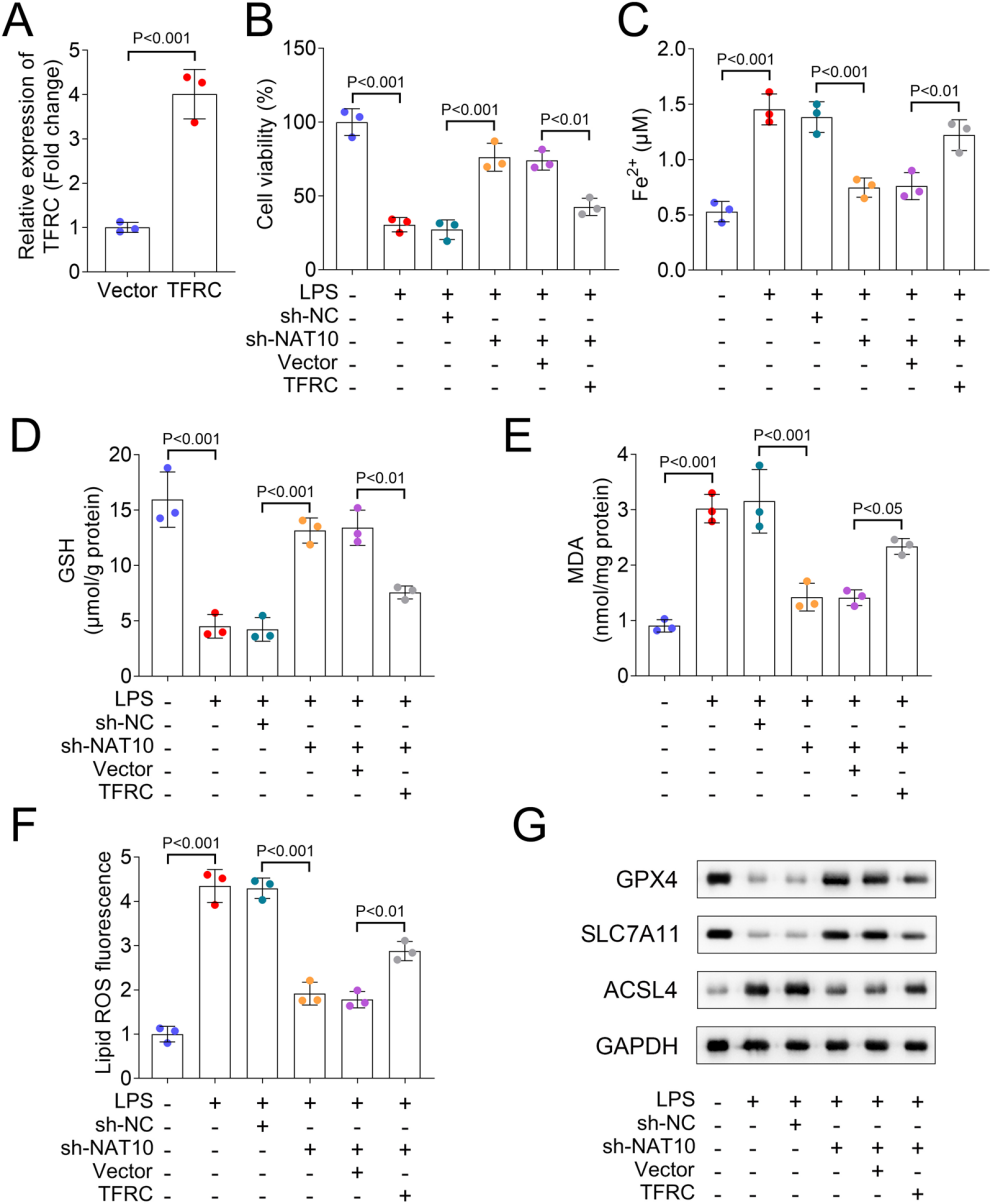
Overexpression of TFRC decreased cell viability and promoted ferroptosis in HULEC-5a cells. **A** The expression of TFRC after TFRC overexpression in HULEC-5a cells was detected by RT-qPCR; **B** The cell viability in each group was analyzed by CCK-8 assay; **C** Fe^2+^, **D** GSH, and **E** MDA concentrations in each group were detected by commercial kits; **F** Flow cytometric analysis of fluorescence intensity of lipid ROS; **G** Western blot was perfomed to assess the protein levels of GPX4, SLC7A11, and ASCL4. CCK-8: cell counting kit-8; RT-qPCR: reverse transcription-polymerase chain reaction; HULEC-5a: human lung microvascular endothelial cell line-5a; GSH: glutathione; MDA: malondialdehyde; GPX4: glutathione peroxidase 4; SLC7A11: solute carrier family 7 member 11; ASCL4: achaete-scute complexlike 4; ROS: reactive oxygen species

In further rescue studies, we transfected TFRC overexpression vector into HULEC-5a cells, and the results showed that the expression of TFRC was upregulated compared with the vector group (Fig. 4A). Besides, compared with the vector group, TFRC overexpression downregulated the cell viability and GSH content and upregulated Fe^2+^ level, MDA concentration, and lipid ROS fluorescence in HULEC-5a cells (Fig. 4B–F). Moreover, in comparison with the vector group, TFRC group decreased GPX4 and SLC7A11 protein levels and increased that of ACSL4 in HULEC-5a cells (Fig. 4G).

### NAT10 inhibition alleviated lung injury in in vivo study

**Fig. 5:**
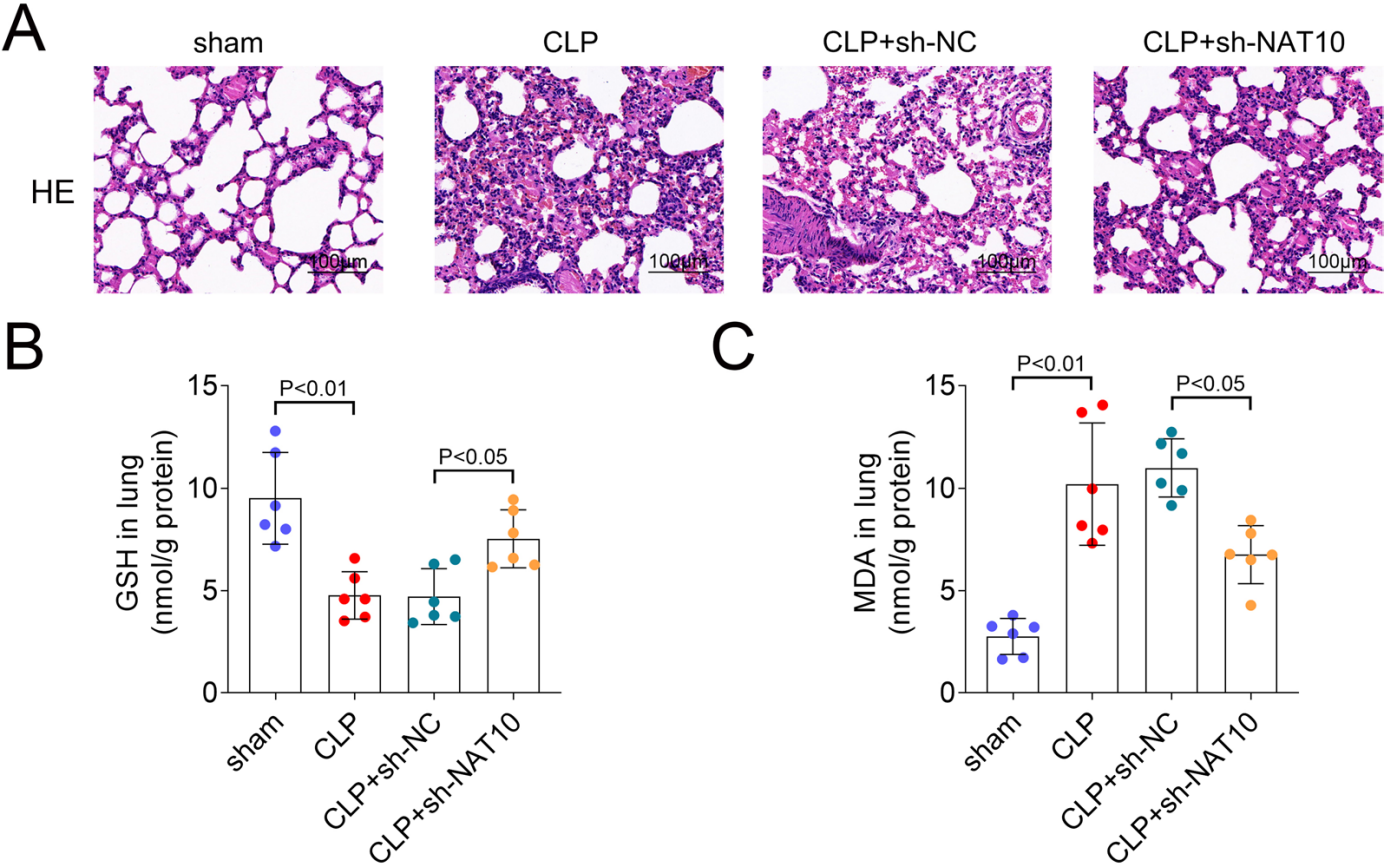
NAT10 inhibition alleviated lung injury in in vivo study. **A** H&E staining was performed to detect the pathological change of lung tissues (scale bar = 100 μm); **B** Detection of GSH, and **C** MDA concentrations in each group. H&E: hematoxylin & eosin; GSH: glutathione; MDA: malondialdehyde

Finally, we established the sepsis-induced ALI rat model to explore the role of NAT10 in vivo. HE staining showed that in the sham group, the alveolar walls of the mice were smooth, the alveolar cavities were free of exudation, and the structure was clear and complete. Besides, a large number of red blood cells observed in the alveolar cavities and inflammatory cells were exuded from the alveolar cavity in the CLP group. The alveolar septa and walls were significantly thickened, and partial alveolar cavity collapsed. Whereas, these pathological symptoms were effectively improved after NAT10 inhibition (Fig. 5A). Furthermore, CLP group rats showed decreased GSH content and increased MDA concentration in lung tissues compared with the sham group. Besides, compared with the sh-NC group, NAT10 knockdown upregulated GSH content and downregulated MDA content (Fig. 5B, C).

## Discussion

Ac^4^C is one of the most frequent RNA epigenetic alterations. NAT10 is the only known ac^4^C “writer” protein. At present, NAT10 is mainly focused on cancer-related research (Wei et al. 2023; Zheng et al. 2022). In the present study, we found that NAT10 was increased in ARDS patients and LPS-treated HULEC-5a cells. Interestingly, a recent study indicates that NAT10 expression is reduced in patients with sepsis and correlated with clinical severity (Zhang et al. 2022a). The reason for this difference may be that we were targeting SPI in this study rather than sepsis alone. In studies of lung-related diseases, NAT10 is significantly increased in lung cancer tissues and pulmonary epithelia exposed to PM_2.5_ (Liu et al. 2023; Shenshen et al. 2023), which was consistent with our results. In addition, NAT10 has also been found to be closely related with other complications of sepsis, such as skeletal muscle atrophy (Wang et al. 2023).

Besides, we found that silencing of NAT10 increased cell viability and suppressed ferroptosis in HULEC-5a cells in the present study. Ferroptosis is a novel form of cell death with prominent features including elevated ROS, lipid peroxidation, iron accumulation, and GSH deprivation. GPX4 and SLC7A11, as a central regulator of ferroptosis, protects cells from ferroptosis by neutralizing lipid peroxides. In contrast, ACSL4 positively regulates ferroptosis (Wu et al. 2019; Chen et al. 2021). Similar to our results, in sepsis-associated ALI, ferroptosis is activated in alveolar epithelial cells, manifested by the changes in GSH, MDA, and serum ferritin levels (Zhang et al. 2022b). Likewise, inducers of pulmonary fibrosis and injury, namely, bleomycin (BLM) and LPS, induced ferroptosis of lung epithelial cells, as evidenced by lipid peroxidation accumulation, iron overload, and increased ROS production (Pei et al. 2022). In addition, serum from coronavirus disease 2019 (COVID-19) non-survivors triggers ferroptosis in endothelial cells, which was manifested by elevated lipid peroxidation levels and decreased expression of GPX4, SLC7A11, and ferritin heavy chain (FTH1) (Jankauskas et al. 2023). Moreover, Mahmood Hassan Dalhat (Dalhat et al. 2023) demonstrates that in NAT10-depleted cancer cells, the expression of essential genes such as SLC7A11, which are associated with ferroptosis, are significantly downregulated. In addition, NAT10 promotes development of colon cancer by affecting ferroptosis (Zheng et al. 2022). Our findings extended the understanding about NAT10 on sepsis-induced diseases, indicating that NAT10 might be a promising therapeutic target.

TFRC is a transmembrane protein that plays a crucial role in regulating iron homeostasis within cells (Moharir et al. 2023). The extracellular domain of TFRC exhibits a strong affinity for di-iron transferrin, which is essential for the effective uptake of iron by cells (Lin et al. 2023). Numerous studies have shed light on the role of TFRC in regulating the ferroptosis sensitivity. (Zhang et al. 2022c) indicate that circular RNA RAPGEF5 (circRAPGEF5) regulates the levels of MDA, lipid ROS and Fe^2+^ by regulating the alternative splicing of TFRC, thereby promoting resistance to ferroptosis. Besides, TFRC knockdown blocked mono-2-ethylhexyl phthalate-induced ferroptosis by decreasing mitochondrial and intracellular levels of Fe^2+^ in Sertoli cells (Zhao et al. 2023). In addition, Yi et al. demonstrate that TFRC upregulation promotes ferroptosis in coxsackievirus (CV) B3 infection via nucleus recruitment of Sp1 transcription factor (Yi et al. 2022). However, the molecular regulation of TFRC in SPI is still unclear. In this study, we found that TFRC was a downstream regulatory target of NAT10-mediated ac^4^C acetylation in HULEC-5a cells. In addition, overexpression of TFRC decreased cell viability and promoted ferroptosis in HULEC-5a cells. Similar with our outcomes, regulation of TFRC-related axis promotes ferroptosis in sepsis-associated encephalopathy (Wei et al. 2022). Besides, in mice with idiopathic pulmonary fibrosis, overexpression of TFRC leads to elevated intracellular Fe^2+^, which promotes fibroblast to myofibroblast transformation (Pei et al. 2022). In lung cancer cells, ferroptosis upregulation suppresses the cell growth by TFRC-related signaling pathway (Huang et al. 2023). Moreover, targeting TFRC attenuates ferroptosis sensitivity in lung cancer (Zhang et al. 2024). In further animal study, we found that NAT10 inhibition alleviated SPI and ferroptosis, which was consistent with our in vitro study.

Taken together, the present research for the first time unveiled that NAT10-mediated ac^4^C acetylation of TFRC regulated ferroptosis, thereby promoting the progression of SPI. These findings indicated that targeting NAT10 may be a potentially effective clinical strategy for SPI treatment. However, there are still some limitations to this study: Firstly, our clinical samples are limited due to the difficulty of clinical sample collection. Besides, we did not study the mechanism in clinical and animal studies. Furthermore, the ac^4^C modification sites of NAT10 on TFRC has not been investigated in this study. These deficiencies will be further explored in our future research.

## Data Availability

The datasets used and/or analysed during the current study are available from the corresponding author on reasonable request.
